# Thiazole as a Promising Scaffold for the Treatment of Schistosomiasis: In Vitro and In Vivo Activity Against Different Developmental Stages of *Schistosoma mansoni*

**DOI:** 10.3390/ph19030420

**Published:** 2026-03-04

**Authors:** João Victor Ritinto da Rocha, Wilza Wanessa Melo França, Arthur Lessa Machado, Lucas Andrade Oliveira Cavalcante, Maria Tairla Viana Gonçalves, Thierry Wesley de Albuquerque Aguiar, Diego Santa Clara Marques, Pedro Henrique do Bomfim Nascimento, Hallysson Douglas Andrade de Araújo, Iranildo José da Cruz Filho, Maria do Carmo Alves de Lima, André de Lima Aires

**Affiliations:** 1 Centro de Ciências Médicas, Programa de Pós-Graduação em Medicina Tropical, Universidade Federal de Pernambuco, Avenida Prof. Moraes Rego, 1235, Cidade Universitária, Recife 50670-501, PE, Brazil; joao.ritinto@ufpe.br (J.V.R.d.R.); lucas.aocavalcante@ufpe.br (L.A.O.C.); tairla.viana@ufpe.br (M.T.V.G.); 2 Instituto Keizo Asami (iLIKA), Universidade Federal de Pernambuco, Avenida Prof. Moraes Rego, 1235, Cidade Universitária, Recife 50670-501, PE, Brazil; arthur.lessa@ufpe.br (A.L.M.); thierry.wesley@ufpe.br (T.W.d.A.A.); hallysson.douglas@ufpe.br (H.D.A.d.A.); 3 Centro de Biociência, Programa de Pós-Graduação em Morfotecnologia, Universidade Federal de Pernambuco, Avenida Prof. Moraes Rego, 1235, Cidade Universitária, Recife 50670-501, PE, Brazil; diego.scmarques@gmail.com (D.S.C.M.); iranildo.cruz@ufpr.br (I.J.d.C.F.); 4 Centro de Biociência, Departamento de Antibióticos, Universidade Federal de Pernambuco, Avenida Prof. Moraes Rego, 1235, Cidade Universitária, Recife 50670-501, PE, Brazil; pedro.bomfim@ufpe.br (P.H.d.B.N.); maria.calima@ufpe.br (M.d.C.A.d.L.); 5 Laboratório de Biotecnologia e Fármacos, Laboratório de Tecnologia de Biomateriais, Centro Acadêmico de Vitória de Santo Antão, Universidade Federal de Pernambuco, Vitória de Santo Antão 55608-680, PE, Brazil

**Keywords:** antiparasitic, schistosomicide, immature stages, thiazole, selectivity index

## Abstract

**Background**: Schistosomiasis affects more than 250 million people, and praziquantel remains the only drug available for treatment; however, its activity is restricted to adult worms. Previously, our group evaluated six thiazole derivatives (**PBT1**–**PBT6**) in vitro against adult *Schistosoma mansoni*, identifying **PBT2**, **PBT5**, and **PBT6** as the most active compounds. The present study aimed to evaluate the in vitro activity of **PBT2**, **PBT5**, and **PBT6** against schistosomula and juvenile worms, as well as their in vivo efficacy against adult *S. mansoni*. **Methods**: Mechanically transformed schistosomula and juvenile worms recovered from mice (21 days post-infection) were incubated with the compounds (12.5–200 μM). Cytotoxicity was assessed using murine splenocytes and peritoneal macrophages exposed to the same concentration range. For in vivo evaluation, infected mice were orally treated with compounds (50, 100, or 200 mg/kg) for five consecutive days. **Results**: All compounds induced 100% mortality in schistosomula and juvenile worms within 3 h of exposure at 100 and 200 μM. Parasite cell viability was markedly reduced (>90%) at concentrations between 50 and 200 μM. The LC_50_ values ranged from 15.3 to 30.9 μM for schistosomula and from 27.8 to 34.9 μM for juvenile worms, with low cytotoxicity observed in mammalian cells (CC_50_ ≥ 193.9 μM). In vivo treatment resulted in significant reductions in fecal egg counts (~80% at 200 mg/kg), total worm burden (~60%), and egg loads in liver and intestinal tissues, in addition to an increased proportion of dead eggs in the intestine. **Conclusions**: The evaluated thiazole derivatives demonstrated potent in vitro activity against immature stages of *S. mansoni* and significant in vivo efficacy against adult parasites, accompanied by favorable changes in key parasitological parameters. These findings reinforce the potential of thiazole-based compounds as promising multistage schistosomicidal candidates.

## 1. Introduction

Schistosomiasis is a helminthic disease caused by blood flukes of the genus *Schistosoma* spp. and affects more than 250 million people across 78 countries and territories worldwide, placing nearly one billion individuals at risk of infection. The disease is responsible for more than 300,000 deaths every year [1,2]. In 2021, schistosomiasis accounted for approximately 1.8 million disability-adjusted life years (DALYs), and its persistence, reflected by high prevalence and incidence rates in endemic regions, continues to represent a major public health challenge despite ongoing control efforts. Beyond the substantial burden on healthcare and security systems, schistosomiasis causes profound socioeconomic and emotional losses [2,3,4,5]. The distribution of schistosomiasis cases and pathogenicity varies according to the *Schistosoma* species, since each one has biological particularities, different intermediate hosts (snails), and distinct epidemiological patterns [1]. Depending on the species, schistosomiasis affects the hepatosplenic and gastrointestinal systems or the urogenital system [1,2].

*S. haematobium* inhabits the venous plexus of the bladder and causes the urogenital form of schistosomiasis, which has the greatest public health impact [1,2,5,6]. According to the World Health Organization, it is the most pathogenic species, accounting for approximately 60–70% of cases worldwide, mainly in Africa and the Middle East [5,6,7]. The infection is characterized by hematuria, bladder fibrosis, lesions of the ureters and kidneys, squamous cell carcinoma of the bladder, and genital manifestations, including testicular pain and hematospermia in men, and abdominal and pelvic pain, dyspareunia, ectopic pregnancy, and infertility in women; it is also associated with increased HIV transmission in co-endemic areas [1,5,6,7]. *S. mansoni*, *S. japonicum*, and *S. intercalatum* are the species responsible for the hepatosplenic and gastrointestinal forms of schistosomiasis [1,6,7]. *S. mansoni* inhabits the hepatic portal system and mesenteric veins and accounts for approximately 30–35% of global schistosomiasis cases, particularly in Africa and South America; it is also the only species endemic in Brazil [4,5,6,7]. The infection typically manifests with abdominal pain, diarrhea, hematochezia, and Katayama syndrome during the acute phase. In chronic and advanced stages, it can progress to hepatosplenomegaly, periportal and intestinal fibrosis, portal hypertension, and ascites [1,2,3].

Despite advances in scientific knowledge, no licensed vaccine for schistosomiasis is currently available, and its clinical development remains uncertain [8]. Consequently, disease control relies exclusively on praziquantel (PZQ), a pyrazylisoquinoline derivative that has been used for more than six decades [5,9]. However, PZQ does not prevent reinfection and shows limited efficacy against early developmental stages, such as schistosomula and juvenile worms, at recommended doses, being predominantly active against adult worms [4,8,10].

The lack of alternative therapeutic options reflects stagnation in pharmacological development, largely due to the high cure rates achieved by PZQ against adult worms, although it is crucial in preventing thousands of deaths. Nevertheless, Amorim et al. [11] warn that reduced PZQ efficacy could lead to a setback in schistosomiasis control. For this reason, the search for new therapeutic alternatives is urgent. Moreover, emerging evidence of *Schistosoma* spp. strains resistant or tolerant to PZQ has been reported [8,9]. In 2021, only 30% of infected individuals had access to PZQ treatment, underscoring its limited availability relative to global demand and reinforcing the urgent need for investment in research and development of new schistosomiasis drugs [2].

In this context, the thiazole nucleus, a heterocycle scaffold containing sulfur and nitrogen atoms, has emerged as a versatile and promising framework in medicinal chemistry for the synthesis of active molecules [12,13,14]. Thiazole derivatives are present in several clinically approved drugs due to their multi-target pharmacophoric groups, such as antimicrobials (sulfathiazole), antifungals (abafungin), antivirals (ritonavir), and antiparasitics (thiabendazole and nitazoxanide) [15,16,17,18]. Thiazole compounds have also been used in preclinical in vitro and in vivo screening of new drugs [12,14] as antioxidants [19], antitumor [20], antibacterial and antibiofilm agents [21], and against *Mycobacterium tuberculosis* [22]. Thiazole compounds have demonstrated in vitro and in vivo activity in antiparasitic studies against *Trypanosoma cruzi* [13,23,24], *Toxoplasma gondii* [25,26], *Plasmodium* spp. [27], *Leishmania* spp. [13], and *Giardia intestinalis*, *Trichomonas vaginalis*, *Leishmania amazonensis*, and *T. cruzi* [28]. In a review, Vajedpour et al. [29] described the promising activity of thiazoles as anthelmintics against nematodes, cestodes, and platyhelminths.

Previously, our group demonstrated that novel thiazole derivatives (**PBT2**, **PBT5**, and **PBT6**) exhibit potent in vitro schistosomicidal activity against adult *S. mansoni* worm pairs, promoting mortality, impaired motility, and reduced cell viability, along with favorable in silico ADMET profiles [10]. The present study aimed to extend the preclinical evaluation of these compounds by investigating their in vitro activity against schistosomula and juvenile worms using mortality, motility, and cell viability scores, and their in vivo efficacy against adult *S. mansoni*, focusing on parasitological load, tissue egg burden in liver and intestinal tissues, and oviposition patterns.

## 2. Results and Discussion

### 2.1. Thiazole Compounds Induce Mortality, Impair Motility, and Reduce Cell Viability in Schistosomula and Juvenile Worms of Schistosoma mansoni

The in vitro susceptibility of *S. mansoni* schistosomula and juvenile worms to **PBT2**, **PBT5**, and **PBT6** is summarized in Table 1 and Table 2, respectively. At all evaluated time points (3, 6, 12, and 24 h), schistosomula and juvenile worms from control groups 1 and 2 exhibited typical motility patterns, characterized by coordinated movements along the body, peristalsis of internal organs, sucker adhesion to the culture plate surface, and preservation of color and tegument integrity, corresponding to score 3 (Table 1 and Table 2). These observations are consistent with those previously described in studies evaluating in vitro screening of schistosomicidal compounds [10,30,31].

All three compounds induced rapid and pronounced schistosomicidal effects. Complete mortality (score 0) of schistosomula and juvenile worms was observed as early as 3 h post-exposure at concentrations of 200 and 100 μM, characterized by the absence of body motility and internal organ peristalsis. After 6 h, **PBT2** and **PBT6** (50 μM) induced 82.9% and 100% mortality in schistosomula, respectively, whereas **PBT5** at the same concentration promoted marked motility reduction to the anterior and/or posterior extremities in 100% of schistosomula (score 1.5).

After 12 h of exposure, **PBT2** and **PBT5** at 50 μM resulted in complete schistosomula mortality, while **PBT6** achieved the same effect at both 50 μM and 25 μM. After 24 h, **PBT2** induced 100% mortality at 25 μM, whereas at 25 and 12.5 μM caused sublethal effects, reducing motility to score 1.5. Against juvenile worms, **PBT6** at 50 μM caused complete mortality within 3 h, while 25 μM reduced motility to score 1 after 24 h. **PBT2** and **PBT5** at 50 μM reduced juvenile worm motility to score 1 in 100% and 66.3% of worms, respectively, within 12 h, with total mortality observed at 24 h.

In contrast, PZQ did not induce mortality in schistosomula or juvenile worms and resulted in reduced motility scores of 1.5 and 1, respectively. These findings corroborate previous reports demonstrating the lack of PZQ activity against immature stages of *S. mansoni*, which limits its efficacy primarily to adult worms [8,9,32]. It is serious that *S. mansoni* in immature stages is refractory to PZQ; in addition to the selection of strains under drug pressure, the parasite undergoes adaptive mechanisms to survive, migrate, and complete its development into adult worms, establishing pathogenicity and the continuity of its biological cycle [4,9].

Previous work by Silva et al. [10] demonstrated that **PBT2**, **PBT5**, and **PBT6** exhibited schistosomicidal activity against adult *S. mansoni* worm pairs, although higher concentrations and longer exposure times were required compared to those observed in the present study. In this study, **PBT2** and **PBT5** caused 100% mortality at 200 µM within 3 h and at 100 µM within 6 h, while **PBT6** at 200 µM promoted 100% mortality only after 12 h and 81.25% at 100 µM within 24 h. In our study, **PBT2**, **PBT5**, and **PBT6** exhibited activity against schistosomula and juvenile worms at 200 μM and 100 μM, achieving mortality within 3 h, while the same effect was observed at 50 μM and 25 μM after 24 h. Furthermore, we observed a marked reduction in motility at 12.5 μM within 24 h, indicating a sublethal effect and suggesting sustained antiparasitic pressure even at low concentrations.

In vitro studies on synthetic compounds targeting immature stages of *S. mansoni* remain limited. Thiosemicarbazone **JF31** and thiazolidinone **JF43** at 200 μM promoted 100% mortality against adult worm pairs and juvenile worms after 24 h and 48 h, respectively [30]. Aryl-thiazoles **NJ05** and **NJ07** at 50 μM caused 75% and 50% mortality against schistosomula, respectively, in addition to approximately 50% mortality against adult worm pairs within 120 h post-exposure [32]. Santiago et al. [33], when evaluating hydrazone, thiosemicarbazone, phthalimide, and thiazole compounds against adult *S. mansoni* worm pairs, reported that activity was exclusive to the thiazoles **LpQM-43**, **LpQM-45**, **LpQM-47**, and **LpQM-14**. **LpQM-45** and **LpQM-14** caused 100% mortality at 100 and 80 μg/mL within 144 and 168 h, respectively, while **LpQM-43** and **LpQM-47** caused 67% and 95% mortality, respectively, in 192 h at 100 μg/mL. Compared to other thiazole-based compounds reported in the literature, **PBT2**, **PBT5**, and **PBT6** exhibited superior potency, inducing rapid lethality at substantially lower concentrations and shorter incubation periods. These findings highlight the therapeutic relevance of thiazole scaffolds as promising candidates for multistage schistosomicidal therapy.

### 2.2. Thiazole Compounds Significantly Reduce Cell Viability of Schistosomula and Juvenile Worms

**Figure 1:**
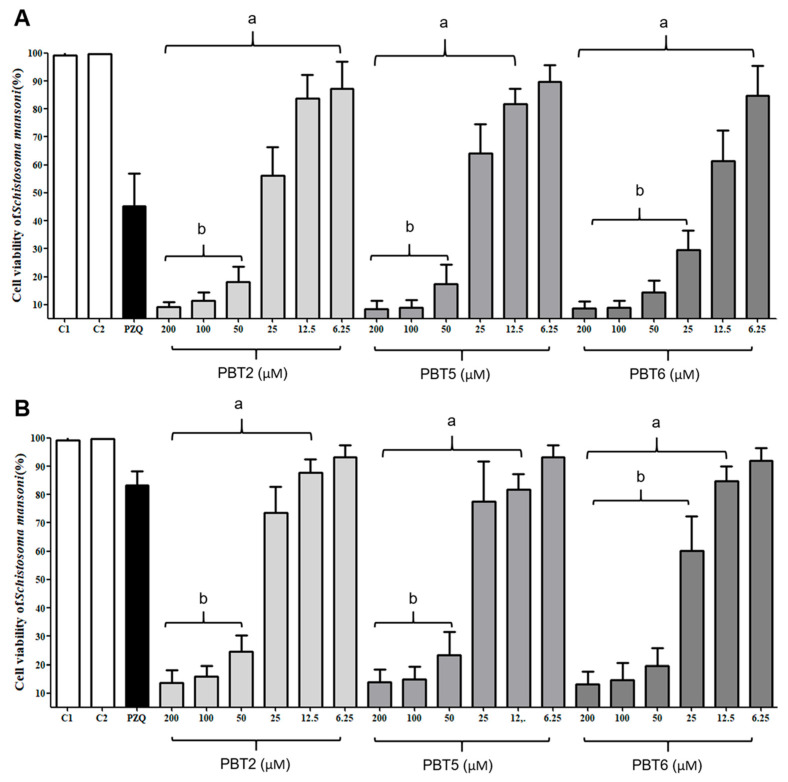
In vitro effect of thiazole compounds (**PBT2**, **PBT5** and **PBT6**) on the cell viability of *Schistosoma mansoni* schistosomula (**A**) and young worms (**B**). Positive controls were treated with praziquantel (PQZ, 10 μM). Viability was expressed as mean ± SD in percentage viability. ^a^ *p* < 0.001 compared to the negative control (C2). ^b^ *p* < 0.001 compared to the positive control PZQ. Control 1 (C1): supplemented RPMI. Control 2 (C2): RPMI supplemented with 1.0% DMSO.

The effects of **PBT2**, **PBT5**, and **PBT6** on parasite cell viability are shown in Figure 1A,B. All compounds induced a concentration-dependent reduction in cell viability in both schistosomula and juvenile worms. In schistosomula, all compounds significantly reduced cell viability (*p* < 0.001), except for **PBT5** at 6.25 µM. At concentrations 200, 100, and 50 μM, cell viability was reduced by more than 90% compared to the negative control (C2, *p* < 0.001; Figure 1A). At 25 μM, **PBT2** and **PBT5** reduced cell viability by 43.7% and 36.0%, respectively, whereas **PBT6** exerted a more pronounced effect, reducing viability by 71.0% at 25 μM and 38.5% at 12.5 μM (*p* < 0.001).

In juvenile worms, all compounds significantly reduced cell viability (*p* < 0.001) at concentrations ranging from 12.5 and 200 µM, with reductions exceeding 80% at concentrations 50 µM, 100 µM, and 200 µM compared to the control (C2). At lower concentrations, **PBT2** reduced cell viability similarly to PZQ in schistosomula; a similar effect was observed in young worms at concentrations of 25 μM and 12.5 μM treated with **PBT2** and **PBT5**. Parasites exposed to PZQ exhibited viability values of 45.2% in schistosomula and 83.2% in juvenile worms, consistent with its limited activity against immature stages (Figure 1A,B). Results are consistent with studies by Araújo et al. [34], Talaam et al. [35], and Silva et al. [31] which highlight that PZQ has a low effect on the cell viability of the immature stage of *S. mansoni*. Against adult worm pairs, Silva et al. [10] reported that **PBT2**, **PBT5**, and **PBT6** promoted a significant reduction (*p* < 0.001) in cell viability, a similar reduction to PZQ, at concentrations of 200 and 100 μM after 120 h.

Triazole compounds are known to interact with numerous cells in different biological targets, reducing or preventing biological activity in the target organism. The tegument of *S. mansoni* is rich in mitochondria and plays a central role in parasite energy metabolism, proliferation, tissue repair, and selective drug uptake [35,36]. Disruption of mitochondrial function is therefore a critical target for schistosomicidal compounds [32,36,37,38,39,40]. The pronounced reduction in parasite viability observed in this study suggests that **PBT2**, **PBT5**, and **PBT6** induce mitochondrial dysfunction, surpassing the effects of PZQ under the evaluated conditions. Thiazole compounds exhibit schistosomicidal activity through multimodal mechanisms involving structural and metabolic alterations [41,42]. Our results indicate that the mitochondrial dysfunction induced by the compounds arises from their interaction with nucleic acid-rich organelles, thereby triggering cytotoxic effects in the worms, a mechanism previously reported for other schistosomicidal candidates [10,11,29,30,32,33,34,35,39,42].

### 2.3. Lethal Concentration, Cytotoxicity, and Selectivity Index

**PBT2**, **PBT5**, and **PBT6** exhibited LC_50_ values of 27.84 ± 3.4, 30.89 ± 2.8, and 15.33 ± 2.2 μM against schistosomula, and 32.85 ± 3.8, 34.92 ± 3.2, and 27.76 ± 2.5 μM against juvenile worms, respectively (Table 3). According to established criteria [37,38], all compounds were classified as active (LC_50_ < 50 μM). These LC_50_ values were notably lower than those previously reported by Silva et al. [10] for adult worm pairs, indicating enhanced efficacy against immature stages. Similar results were obtained by Oliveira et al. [39], Pereira et al. [32], and Oliveira Barbosa et al. [40] regarding the viability of *S. mansoni* exposed to different thiazole compounds. Series of phthalimido-thiazole compounds against *S. mansoni* adult worms exhibited LC_50_ values ranging from 138.09 to 302.97 μM [39] and 50.6 to 305.6 μM [40]. Compounds with improved lipophilicity, stability and permeability ensure better access to the target, and the interference in more than one biological pathway of the parasite and the reduction in its metabolic compensation capacity result in a lower LC_50_ [41], as demonstrated by LC_50_ values of 49.36–114.18 μM [42]. Thiosemicarbazone and thiazole compounds promoted LC_50_ values ranging from 19.97 to 84.13 μM against *S. mansoni* pairs of adult worms [33]. The identification of active compounds in multiple stages of *S. mansoni* may overcome the limitation of PZQ against immature forms and expand the effectiveness of chemotherapy in endemic areas [8].

The absence or low cytotoxicity in mammalian cells and high parasite selectivity are essential requirements for schistosomicidal candidates [8,10,42]. In the studies by Silva et al. [10], using immortalized cell lines, **PBT2**, **PBT5**, and **PBT6** showed low cytotoxicity with CC_50_ ranging from 107.05 μM to >400 μM in RAW 264.7 macrophages, from 140.01 μM to >400 μM in fibroblasts, from 161.88 μM to >400 μM in Vero cells, and >400 μM for HepG2 cells. Furthermore, the three compounds evaluated did not promote hemolysis. In contrast to the absence of cytotoxicity of **PBT2**, **PBT5**, and **PBT6** in HepG2 cells (>400 μM), thiazole series exhibited substantially lower CC_50_ in this cell line, ranging from 6.78 to 40.33 μM [43] and from 13.92 to 117.78 μM [44].

CC_50_ values for thiazoles are predominantly concentrated in immortalized or tumor cell lines, being limited in primary cells. Primary cells preserve characteristics and functions more similar to in vivo tissue than immortalized cell lines, improving the prediction of drug toxicity and safety in preclinical evaluations for subsequent in vivo trials [45]. Cytotoxicity assays using primary murine splenocytes and peritoneal macrophages revealed low toxicity for all compounds, with CC_50_ values ranging from 193.9 to 297.3 μM in splenocytes and ≥277.5 μM in macrophages (Table 3). In contrast, PZQ exhibited marked cytotoxicity in splenocytes (CC_50_ = 3.2 ± 0.8 μM). None of the compounds met the criteria for cytotoxicity (CC_50_ > 100 μM), indicating a favorable safety profile [46].

The selectivity index (SI) values ranged from 5.84 in juvenile worms to greater than 26 in schistosomula. High selectivity indices are indicative of a favorable safety profile and therapeutic window and reinforce the potential of these thiazole derivatives as promising schistosomicidal candidates [38]. **PBT6** promoted lower CL_50_ and higher CC_50_ compared to the other compounds, resulting in a higher SI, indicating better antiparasitic activity associated with lower cytotoxicity. The 4-phenyl-2-aminothiazole **GPQF-108** presented an LC_50_ of 29.44 μM against adult worms and CC_50_ of 179.44 in Vero cells, resulting in an SI of 6.09 [11]. Phthalimido-thiazoles showed low cytotoxicity on Vero cells (CC50 of 112.9 to 1582.9 μM) [47] and on macrophage J774 (CC50 of 125.6 μM to 1582.9 μM) [40]. The diversity of these results shows that thiazoles stand out as versatile scaffolds whose structural variations modulate schistosomicidal activity and demonstrate their potential as a promising platform for the rational development of new schistosomicides [32].

### 2.4. The Compounds Reduce Fecal Egg Output, Adult Worm Burden, Tissue Egg Deposition, and Alter the Oviposition Pattern in Mice Infected with Schistosoma mansoni

The Kato–Katz technique is widely used for the diagnosis of schistosomiasis and for evaluating parasitological cure, as it enables estimation of infection intensity and classification of endemic areas for mass drug administration [48]. Prior to treatment, significant differences were observed among the experimental groups regarding the number of eggs per gram of feces (EPG) (*p* > 0.05), with values ranging from 322.7 ± 53.8 to 357.3 ± 50.1, indicating homogeneous infection levels across groups.

**Figure 2:**
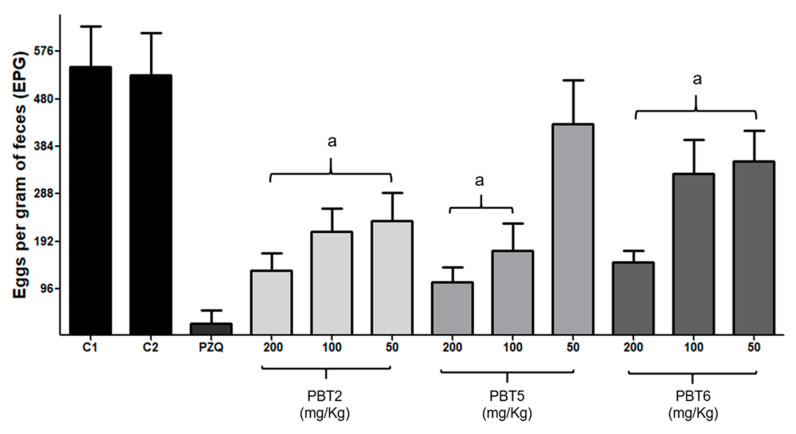
Effect of thiazole compounds (**PBT2**, **PBT5**, and **PBT6**) and praziquantel (PZQ) on fecal egg counts (EPG) in *S. mansoni*-infected mice. Data are presented as mean ± SD. ^a^ *p* < 0.001 versus negative controls (C1 and C2). The positive control worms were treated with praziquantel (PQZ, 10 μM).

Following treatment, except for the 50 mg/kg dose of **PBT5**, all compounds induced a significant reduction in EPG values (*p* < 0.001), reaching approximately 80% reduction at the 200 mg/kg dose (Figure 2). These findings indicate a strong potential of the compounds to reduce environmental contamination and interrupt the biological cycle of *S. mansoni* [1,48,49]. PZQ reduced EPG values by 95.1% compared with the control group, consistent with previous studies [50,51,52]. Amorim et al. [11] demonstrated that among 17 4-Phenyl-2-Aminothiazole derivatives, GPQF-108 promoted a 56% reduction in fecal egg counts following a single oral dose of 400 mg/kg. A significant decrease in oviposition was observed by Pereira et al. [32].

All three compounds reduced total worm burden, female worm counts, and egg deposition in hepatic and intestinal tissues in a dose-dependent manner (Table 4). Treatment with 200 mg/kg resulted in an approximately 60% reduction (*p* < 0.001) in both total and female worm burdens, suggesting comparable activity against male and female parasites. These findings contrast with in vitro and in vivo studies reporting greater susceptibility of male worms, whether paired and unpaired [53,54]. PZQ reduced total worm burden by 99%, corroborating results reported by Aires et al. [51]. Except for the 50 mg/kg dose of **PBT5**, all doses of **PBT2**, **PBT5**, and **PBT6** significantly reduced egg counts in liver and intestinal tissue (*p* < 0.001), with reductions ranging from 34.41 to 59.41% and 29.2–61.5%, respectively, compared with the control group (Table 4).

Understanding parasite biology is essential for the development of new antischistosomal drugs, and analysis of the oviposition pattern through oogram evaluation represents a key parasitological parameter [55]. The oogram is considered altered when one or more developmental stages are absent and modified when significant changes occur in the relative proportions of egg stages. In intestinal tissue samples from control groups 1 and 2, *S. mansoni* eggs were observed at all developmental stages (immature [I–IV], mature, and dead), with a percentage distribution consistent with those previously reported in the literature [31], indicating preserved reproductive dynamics of the worm pairs.

In contrast, treatment with **PBT2**, **PBT5**, and **PBT6** induced significant and dose-dependent alterations in the oviposition pattern, characterized primarily by a reduction in immature eggs and a marked increase in dead eggs (*p* < 0.001), as shown in Table 5. The 200 mg/kg dose yielded the highest proportions of dead eggs, reaching 56.2 ± 7.08%, 53.2 ± 2.16%, and 59.4 ± 6.6% for **PBT2**, **PBT5**, and **PBT6**, respectively, with statistically significant differences compared with the 100 and 50 mg/kg doses. These findings demonstrate that higher compounds doses potentiate their effects on the oviposition pattern of *S. mansoni*. Treatment with PZQ resulted in 95.8% egg mortality in intestinal tissue, a significant reduction in mature eggs (4.2%), and the complete absence of immature eggs.

The activity of PBT compounds on the oogram is particularly relevant, as it indicates schistosomicidal effects on egg viability within intestinal tissue. Reductions in immature and mature eggs may be partially attributed to decreased fecundity of surviving worm pairs and/or delayed embryonic development following oviposition. Conversely, the increase in dead eggs suggests a direct ovicidal effect, comparable to that observed with PZQ.

Amorim et al. [11] reported that **GPQF-108**, administered as a single oral dose of 400 mg/kg, reduced total *S. mansoni* worm burden by 53.74% (*p* < 0.01) and selectively modifying the oogram by reducing immature eggs (38.72%, *p* < 0.05), without affecting mature or dead egg viability. From nine phthalimido-thiazole compounds, only compound **2m** demonstrated biosafety and schistosomicidal activity in vitro against adult *S. mansoni* [40]. In vivo, compound **2m** reduced worm burden by 94.6% following five consecutive days of treatment at 200 mg/kg/day and by 75.6% after a single oral dose of 400 mg/kg. However, neither regimen produced significant reductions in hepatic and intestinal egg loads, nor did they modify the oviposition pattern. Among six phthalimido-thiazoles (**2b**–**2j**), only compound **2i** exhibited in vitro activity after 144 h and advanced to in vivo evaluation in *S. mansoni*-infected mice. Treatment with **2i** at 400 mg/kg for five consecutive days significantly reduced worm burden by 81.25% and 69.2% (*p* < 0.05) following oral and intraperitoneal administration, respectively, with no significant differences between routes [42]. In that study, compound **2i** also reduced intestinal egg load (~45%, *p* < 0.05), without affecting hepatic egg counts, and altered the oogram by decreasing mature (23.7%) and immature (22.5%) eggs while increasing dead eggs (53.8%).

**Figure 3:**
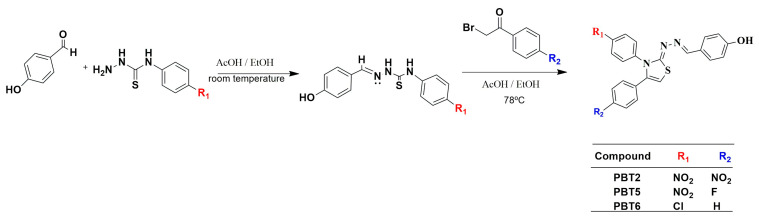
Thiazole compounds synthesis scheme.

Thus far, the mechanism by which thiazole compounds act on *S. mansoni* has not been fully elucidated, possibly due to their structural diversity and the versatility of the thiazole nucleus [30,32,33,56]. In the present study, although **PBT2**, **PBT5**, and **PBT6** exhibited similar schistosomicidal activity in vitro and in vivo, these compounds differ in terms of the substituents on the thiazole ring (–NO_2_, –Cl, –F, and –H; Figure 3). The biological activity of nitroaromatic compounds involves the reductive biotransformation of the nitro group, generating reactive intermediates such as nitro, nitroso, and hydroxylamine radicals, through the generation of reactive oxygen species and depletion of cellular antioxidant systems. These metabolites can react with iron or thiol groups in proteins and other macromolecules, leading to the inactivation of enzymes essential for mitochondrial respiration and DNA replication, promoting the death of the parasite [32,56]. This mechanism is consistent with that described for clinically used nitro-heterocyclic drugs, such as metronidazole, tinidazole, secnidazole, benznidazole, and nifurtimox, whose antiparasitic efficacy critically depends on the reduction in the nitro group and the subsequent formation of cytotoxic metabolites [56,57].

The introduction of halogens into bioactive scaffolds is a well-established strategy in medicinal chemistry to enhance lipophilicity. Halogenated substituents such as chlorine and fluorine can increase passive permeability across biological membranes, as well as promote intermolecular interactions, including hydrophobic contacts and halogen-acceptor bonds (halogen bonding), increasing affinity and selectivity for protein targets and the bioavailability [58,59]. *S. mansoni* possesses a tegumental surface rich in lipids; therefore, increased lipophilicity may facilitate compound permeation through the tegument, enhancing access to intracellular targets. Modulation of tegumental permeability represents a relevant pharmacological mechanism, as exemplified by PZQ, which increases Ca^2+^ permeability in *S. mansoni*. Although it has been used since the 1970s and investigated in numerous studies, the exact mechanism of action of PZQ has not yet been fully elucidated. However, it is known that PZQ acts on Ca^2+^ homeostasis in the integument and is responsible for causing spasms, damage to the integument, and muscular paralysis, leading to the death of adult worms [4,5,9].

**Figure 4:**
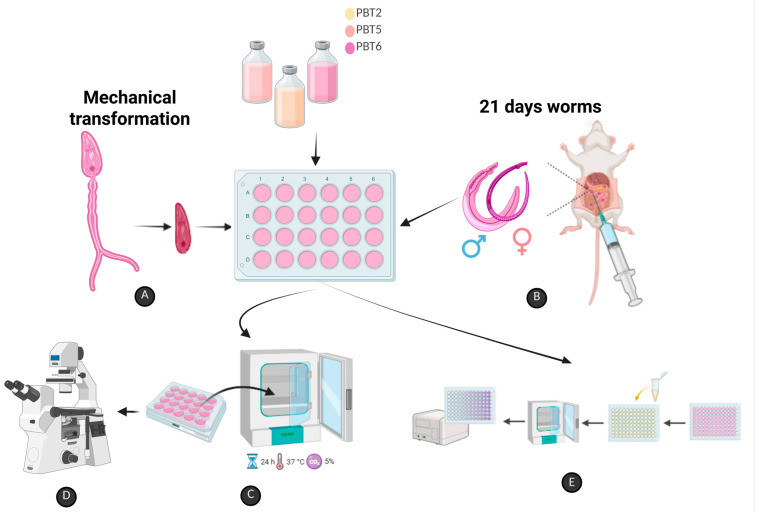
Methodological scheme of in vitro studies. (**A**) Mechanical transformation of cercariae into *Schistosoma mansoni* schistosomes; (**B**) Perfusion of the hepatic portal system for recovery of young worms; (**C**) Plating and incubation (24 h, 37 °C, 5% CO_2_) of schistosomes and young worms with **PBT2**, **PBT5** and **PBT6** compounds; (**D**) Visualization by inverted microscope for evaluation of motility and mortality scores; and (**E**) Evaluation of the cytotoxicity of schistosomes and young worms after incubation with the compounds. Created in BioRender. França, W.W.M. (2026), accessed on 1 February 2026. https://BioRender.com/pcecbz5.

No significant differences were observed between control groups 1 and 2 (C1 and C2; *p* > 0.05) regarding fecal egg counts (Figure 4), worm burden (total and female), hepatic and intestinal egg loads (Table 4), or oviposition pattern (Table 5). These findings confirm that Cremophor^®^ (1%), used as an excipient, exhibited no schistosomicidal activity, supporting its use solely as a solvent [40,54,60] and allowing the observed effects to be attributed exclusively to the evaluated compounds.

## 3. Materials and Methods

### 3.1. Reagents and Solvents

The following commercial reagents and solvents were used: 2-bromoacetophenone (Merck, Rahway, NJ, USA, CAS 70-11-1), 2-bromo-4-nitroacetophenone (Merck, Rahway, NJ, USA, CAS 2227-64-7), 2-bromo-4-chloroacetophenone (Merck, Rahway, NJ, USA, CAS 536-38-9), 2,4-dibromoacetophenone, 2-chloro-4-fluoroacetophenone (Merck, Rahway, NJ, USA, CAS 456-04-2), 4-nitrophenyl isothiocyanate (Merck, Rahway, NJ, USA, CAS 2131-61-5), 4-chlorophenyl isothiocyanate (Merck, Rahway, NJ, USA, CAS 2131-55-7), 4-hydroxybenzaldehyde (Merck, Rahway, NJ, USA, CAS 123-08-0), hydrazine solution (Merck, Rahway, NJ, USA, CAS 302-01-2). Sodium chloride (Merck, Rahway, NJ, USA, CAS 7647-14-5), praziquantel (Merck, Rahway, NJ, USA, CAS 55268-74-1) and Cremophor^®^ (Sigma, Ronkonkoma, NY, USA, C5135-500G). All analytical grade and cell culture reagents were obtained from Sigma Chemical Co., St. Louis, MO, USA.

### 3.2. Synthesis of Thiazole Compounds

Thiazole derivates were synthesized and supplied by the Laboratory of Chemistry and Therapeutic Innovation (LQIT) of the Department of Antibiotics at the UFPE, in Recife, Pernambuco, Brazil. Compound synthesis was performed in three stages following previously described methodologies [61,62,63] with minor modifications. In the first stage, thiosemicarbazides were prepared through a nucleophilic addition reaction between hydrazine and either 4-nitrophenyl isothiocyanate or 4-chlorophenyl isothiocyanate in dichloromethane at room temperature under magnetic stirring for 1 h. In the second stage, thiosemicarbazones were synthesized by reacting the thiosemicarbazides with 4-hydroxybenzaldehyde in absolute ethanol in the presence of acetic acid as a catalyst, under magnetic stirring at room temperature. In the third step, thiazole derivatives were obtained through cyclization of thiosemicarbazones with substituted acetophenones bearing phenyl, 4-chlorophenyl, or 4-nitrophenyl groups, yielding the compounds **PBT2**, **PBT5**, and **PBT6**. The synthetic route is illustrated in Figure 3.

### 3.3. Ethics Committee and Animals

All experimental procedures involving animals were conducted in accordance with the guidelines of the National Council for the Control of Animal Experimentation (CONCEA) and approved by the Ethics Committee on Animal Experimentation (CEUA) of the Center for Biosciences of UFPE, Brazil, (process number 0085/2022 and 0029/2025). Animal studies were reported in accordance with the guidelines “Animal research: reporting of in vivo experiments” (ARRIVE). Male BALB/c mice (8 weeks old, ~25 ± 2 g) and female Swiss Webster mice (35 days old, ~30 ± 2 g) were obtained from and maintained at the animal facility of the Keizo Asami Institute of UFPE (iLIKA-UFPE, Recife, Brazil) under standardized conditions (23 ± 2 °C, 40–50% relative humidity, 12 h light/dark cycle), with free access to water and Labina^®^ chow. *S. mansoni* (BH strain, Belo Horizonte, Minas Gerais, Brazil) has been maintained through successive passages in *Biomphalaria glabrata* snails (SLM strain, São Lourenço da Mata, Pernambuco, Brasil) and Swiss Webster mice at the Academic Area of Tropical Medicine and iLIKA at UFPE. Snails were housed in plastic tanks (50 × 23 × 17 cm) containing filtered and dechlorinated water (20 L), renewed weekly, and fed daily with fresh lettuce leaves (*Lactuca sativa* L.), under controlled temperature (25 ± 2 °C) and a 12 h light/dark photoperiod.

### 3.4. Infection of Biomphalaria glabrata and Collection of S. mansoni Cercariae

*Schistosoma mansoni* eggs were obtained from the feces of experimentally infected mice using the spontaneous sedimentation technique [64]. After repeated washings, the sediment was distributed into Petri dishes and exposed to artificial light (60 W, Lightex, model A5570, Sofia, Bulgaria) to induce miracidia hatching. Using a stereomicroscope, snails were individually infected with five miracidia in 24-well culture plates (TPP-Techno Plastic Products, Trasadingen, Switzerland) and exposed to artificial light for 4 h. Subsequently, snails were transferred to plastic tanks in a light-protected environment. After 35 days post-infection, snails (*n* = 80) were exposed to artificial light (60 W) for 2 h to stimulate cercarial shedding.

### 3.5. In Vitro Assay Against S. mansoni Schistosomula

Cercariae of *S. mansoni* were mechanically transformed into schistosomula as previously described (Figure 4A) [34,65]. Schistosomula were washed four times in RPMI 1640 medium (Sigma Aldrich, St. Louis, MO, USA) supplemented (20 mM HEPES, 100 μg/mL penicillin, 100 μg/mL streptomycin and 10% fetal bovine serum) and incubated at 37 °C in a humidified atmosphere containing 5% CO_2_ for 5 h to allow adaptation.

Subsequently, 70 schistosomula were distributed per well in 24-well culture plates and exposed to **PBT2**, **PBT5**, or **PBT6** diluted in 1% Dimethyl sulfoxide (DMSO) to obtain final concentrations of 200, 100, 50, 25, 12.5, and 6.25 μM (final volume: 2 mL/well). Under the same experimental conditions, negative controls consisting of parasites incubated in supplemented RPMI 1640 alone or RPMI 1640 containing 1% DMSOs schistosomula were incubated only with supplemented RPMI 1640 medium or RPMI. The positive control group was treated with PZQ (10 μM). Two independent experiments were performed in duplicate.

### 3.6. In Vitro Assay Against Juvenile S. mansoni Worms

Five mice were infected caudally with 3000 cercariae and euthanized by cervical dislocation on day 21 post-infection for aseptic recovery of juvenile worms through perfusion of the portal-hepatic system with sterile saline solution (Figure 4B) (0.9% *w*/*v* NaCl) [66]. Recovered worms were transferred to Petri dishes containing supplemented RPMI 1640 medium, washed four times, and incubated (5% CO_2_ at 37 °C) for 2 h to allow adaptation (Figure 4C).

Juvenile worms (*n* = 30) were distributed into 24-well culture plates and exposed to **PBT2**, **PBT5**, or **PBT6** diluted in 1% DMSO to final concentrations of 200, 100, 50, 25, 12.5, and 6.25 μM (final volume: 2 mL/well). Under the same experimental conditions, negative control groups consisted of juvenile worms incubated in supplemented RPMI 1640 alone or supplemented RPMI 1640 containing 1% DMSO. The positive control group was treated with 10 μM of PZQ. Experiments were performed in quadruplicate and repeated independently twice, totaling 120 juvenile worms per concentration.

### 3.7. In Vitro Schistosomicidal Criteria Against Schistosomula and Juvenile Worms

#### 3.7.1. Motility and Survival

Alterations in motility and survival of schistosomula and juvenile worms were evaluated using an inverted microscope (LEICA DM IL Wetzlar, Germany) at 3, 6, 12, and 24 h post-exposure (Figure 4D). Schistosomula motility and survival were scored according to Araújo et al. [34] using a three-point scale: score 3 indicated typical movements along the body, internal organs peristalsis, sucker adhesion, and preserved color and tegument; score 1.5 indicated reduced body motility or movement restricted to extremities with absence of sucker adhesion; score 0 indicated complete absence of movements with or without tegumental alterations.

Juvenile worms were classified using a four-point scale according to Araújo et al. [34] as follows: score 3 indicated typical movements along the body, internal organs peristalsis with sucker adhesion; score 2 indicated reduced motility and peristalsis; score 1 indicated movement restricted to posterior and/or anterior extremities; and score 0 indicated complete absence of movement with or without tegumental alterations. Mortality was defined as score 0 when no movement was observed within a 2 min observation period.

#### 3.7.2. Cell Viability Assay

Cell viability was assessed using 3-(4,5-dimethylthiazol-2-yl)-2,5-diphenyltetrazolium bromide (MTT) assay (Figure 4E) (Sigma-Aldrich, St. Louis, MO, USA), as described previously [34,67,68]. After the final observation time, culture medium was aspirated from the well, and parasites were washed with sterile saline solution (0.9% *w*/*v* NaCl). Subsequently, 20 schistosomula or six juvenile worms were transferred to 96-well plates containing 100 μL of MTT solution (5 mg/mL in PBS) per well and incubated for 30 min at 37 °C in the absence of light.

The MTT solution was then replaced with 200 μL of DMSO, and plates were agitated for 1 h at room temperature to solubilize the formazan crystals. Absorbance was measured at 550 nm using a microplate reader (M680, Bio Rad Laboratories, Inc., Hercules, CA, USA). MTT yellow is reduced to formazan purple by a variety of mitochondrial and cytosolic enzymes that are functional in viable cells. Worms from positive and negative control groups were subjected to the same procedure. Assays were performed in sextuplicate in two independent experiments, and results are expressed as mean ± standard deviation (SD) of percentage cell viability worms. The lethal concentration for 50% of parasites (LC_50_) was calculated by nonlinear regression analysis of dose–response curves using GraphPad Prism version 6.0 for Windows (GraphPad Software, San Diego, CA, USA).

### 3.8. Cytotoxicity Assay in Murine Peritoneal Macrophages and Splenocytes

Five male BALB/c mice (8 weeks old, ~25 ± 2 g) were used for the collection of perito-neal macrophages and splenocytes, as described previously [69,70,71]. Peritoneal macrophages were collected by peritoneal lavage using 5 mL of RPMI 1640. Splenocytes were obtained from aseptically removed spleens, which were mechanically dissociated in RPMI 1640 medium, followed by erythrocyte lysis with sterile distilled water.

Cells were centrifuged, resuspended in supplemented RPMI 1640 medium (10% FBS, 1% antibiotics (streptomycin + penicillin)), and counted using a Neubauer chamber (Biocentrix, São Paulo, Brazil) with trypan blue exclusion. Cells were seeded in 96-well plates at a density of 1 × 10^6^ cells/well and incubated (37 °C, 5% CO_2_). After 24 h, culture medium was replaced with fresh medium containing **PBT2**, **PBT5**, or **PBT6** at concentrations of 6.25–400 μM solubilized in 1% DMSO. The cells were incubated for an additional 48 h at 37 °C in 5% CO_2_.

After incubation, cells were washed and incubated in fresh culture medium without phenol red containing MTT (5 mg/mL) to a final volume of 200 μL/well for 3 h at 37 °C in the absence of light. The culture medium with MTT was then replaced with DMSO (100 μL/well) to solubilize the formazan derived from MTT reduction, and absorbance was measured spectrophotometrically at 540 nm. Control groups consisted of cells exposed only to supplemented RMPI 1640, supplemented RPMI 1640 with 1% DMSO, or 10 μL of PZQ. The cell viability of each concentration was calculated from the absorbances obtained by reading the plate, using the following equation:

$$Viability\;\%=\frac{Absorbance\;(treated\;cells)-(Absorbance\;blank)}{Absorbance\;(Negative\;control)-(Absorbance\;blank)}\times 100$$

Cytotoxic concentration values reducing cell viability by 50% (CC_50_) were calculated by nonlinear dose–response regression in a 95% confidence interval using GraphPad Prism 5.0 software.

### 3.9. In Vitro Selectivity Index

The selectivity index (SI) was calculated by the ratio between the cytotoxicity in mammalian cells (CC_50_) and antiparasitic activity (LC_50_) against schistosomula and juvenile worms, as previously described [38].

### 3.10. Experimental Infection and Therapeutic Protocol

**Figure 5:**
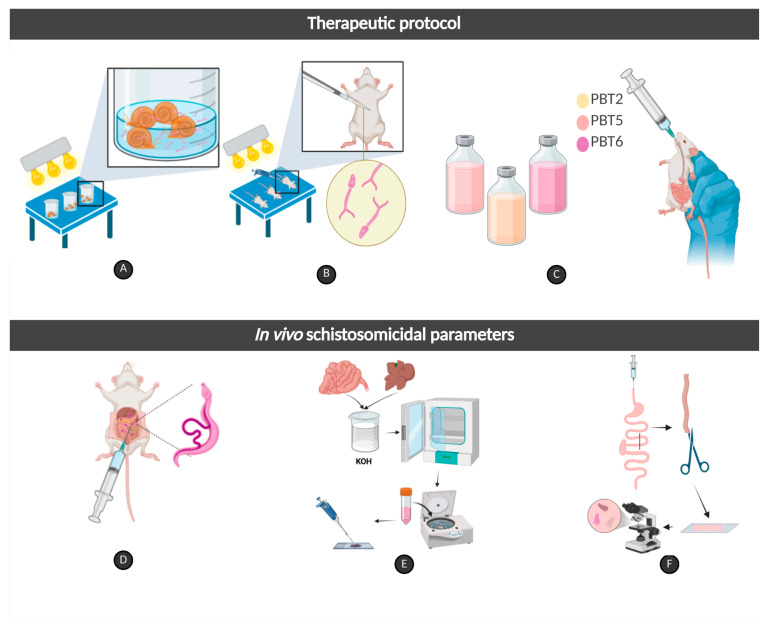
Methodological scheme of in vivo studies. (**A**) Exposure of *Biomphalaria glabrata* for shedding of *Schistosoma mansoni* cercariae; (**B**) Percutaneous infection of mice with *S. mansoni* cercariae; (**C**) Oral administration of **PBT2**, **PBT5**, or **PBT6** compounds; (**D**) Perfusion of the hepatic portal system and mesenteric vessels for recovery of adult *S. mansoni* worms; (**E**) Digestion of hepatic and intestinal tissue in KOH for quantification of eggs in the tissues; and (**F**) Oogram method for evaluating the oviposition pattern. Created in BioRender. França, W.W.M. (2026), accessed on 1 February 2026. https://BioRender.com/d0nc0j5.

A total of 120 mice were anesthetized (ketamine-xylazine (100 mg/kg + 15 mg/kg)) and percutaneously infected with 80 *S. mansoni* cercariae (Figure 5A,B), according to Olivier and Stirewalt [68]. Animals were randomly distributed into 12 experimental groups (*n* = 10/group) according to the therapeutic protocol, namely

- Control 1 = sterile saline solution;
- Control 2 = 1% Cremophor^®^ in sterile saline solution;
- PZQ = 50 mg/kg;
- **PBT2** = 200 mg/kg, 100 mg/kg or 50 mg/kg;
- **PBT5** = 200 mg/kg, 100 mg/kg or 50 mg/kg;
- **PBT6** = 200 mg/kg, 100 mg/kg or 50 mg/kg.

PZQ and the compounds were solubilized in 1% Cremophor^®^ in sterile saline solution (0.9% *w*/*v* NaCl). A dose of 50 mg/kg of PZQ was adopted according to Aires et al. [63], while the doses of the compounds were chosen based on in silico pharmacokinetic and pharmacodynamic prediction [10] and on the evaluation of acute oral toxicity and in vivo schistosomicidal activity of thiazole compounds [40]. Treatments were carried out for five consecutive days starting on the 45th day of infection. The animals were fasted for 60 min, and the treatment was performed via esophageal gavage with a final volume of 300 µL (Figure 5C). The Control 1 and Control 2 groups were maintained under the same rearing and handling conditions. All animals were euthanized on day 55 post-infection by cervical displacement for parasitological analysis.

### 3.11. In Vivo Parasitological Parameters

#### 3.11.1. Adult Worm Recovery

Adult worms were recovered by perfusion of the portal-hepatic system and mesenteric vessels with sterile saline solution (0.9% *w*/*v* NaCl) and counted using a stereomicroscope. Worms were classified as paired or males (Figure 5D), according to Smithers and Terry [72]. Percentage reduction in worm burden after treatment was calculated according to the following equation:

% reduction = C − V/C × 100

where C = the mean number of worms recovered from infected, untreated animals and V = the mean number of worms recovered from treated animals.

#### 3.11.2. Number of Eggs in Liver and Intestinal Tissues

Liver and intestinal tissues were individually processed using the KOH digestion technique (Figure 5E) [73]. Egg counts were expressed as the number of eggs per gram of tissue, and the percentage reduction were calculated.

#### 3.11.3. Oviposition Pattern

Samples from the mid-small intestine (3 cm) were collected to assess developmental stages of *S. mansoni* eggs (Figure 5F), as described by Pellegrino et al. [74]. The tissue was sectioned longitudinally, washed in sterile saline solution, gently dried with filter paper, and subsequently mounted between a slide and coverslip to obtain thin preparations. The slides were examined under an optical microscope for classification and percentage quantification of egg developmental stages: immature (stages I, II, III, and IV), mature, or dead.

### 3.12. Statistical Analysis

GraphPad Prism 5.0 (GraphPad Software, San Diego, CA, USA) was used for statistical analysis. Results were expressed as mean ± standard deviation (SD). Analysis of variance (ANOVA) was used to compare the different experimental groups. When ANOVA revealed a significant difference, the Bonferroni post hoc test was used to identify the difference between the groups. Differences were considered significant when *p* < 0.05.

## 4. Conclusions

In the search for novel antischistosomal drugs, in vitro preclinical screening followed by in vivo evaluation is essential to determine the biological potential and safety of candidate compounds, thereby reducing time, effort, and costs prior to more complex experimental stages. Our findings reinforce the relevance of thiazole derivates as promising schistosomicidal agents.

In vitro, PBT compounds induced rapid and complete mortality, as well as pronounced motility alterations in schistosomula and juvenile worms at low concentrations, within short exposure times, and with high selectivity indices, without inducing significant cytotoxicity in mammalian cells. In vivo, treatment with PBT compounds resulted in marked reductions in total and female worm burdens, the number of eggs per gram of feces and egg deposition in hepatic and intestinal tissues, in addition to significantly altering the oviposition pattern by increasing the proportion of dead eggs.

Although the evaluated compounds exhibit low aqueous solubility and limited oral absorption, the results obtained herein provide a strong rationale for the development of pharmaceutical delivery aimed at improving their pharmacokinetic and pharmacodynamic profiles. Such strategies may enhance the antiparasitic efficacy of these compounds against *S. mansoni* and further strengthen their translational potential as candidates for the development of new schistosomicidal therapies.

## Figures and Tables

**Table 1:** Motility scores of schistosomula, treated with praziquantel (PZQ) or thiazole compounds (**PBT2**, **PBT5**, or **PBT6**) after 3, 6, 12, and 24 h of incubation.

Experimental Groups	Mean ± Standard Deviation and Percentage (%) of Young Worms in Motility Scores After Incubation											
	3 h			6 h			12 h			24 h		
	0	1.5	3	0	1.5	3	0	1.5	3	0	1.5	3
Control 1			70 ± 0.0 (100 %)			70 ± 0.0 (100 %)			70 ± 0.0 (100 %)			70 ± 0.0 (100 %)
Control 2			70 ± 0.0 (100%)			70 ± 0.0 (100%)			70 ± 0.0 (100%)			70 ± 0.0 (100%)
PZQ												
10 µM		70 ± 0.0 (100%)			70 ± 0.0 (100%)			70 ± 0.0 (100%)			70 ± 0.0 (100%)	
**PBT2**												
200 μM	70 ± 0.0 (100%)			70 ± 0.0 (100%)			70 ± 0.0 (100%)			70 ± 0.0 (100%)		
100 μM	70 ± 0.0 (100%)			70 ± 0.0 (100%)			70 ± 0.0 (100%)			70 ± 0.0 (100%)		
50 μM		70 ± 0.0 (100%)		56 ± 8.0 (82.9%)	14 ± 3.0 (17.1%)		70 ± 0.0 (100%)			70 ± 0.0 (100%)		
25 μM			70 ± 0.0 (100%)		33 ± 3.5 (47.2%)	37 ± 2.0 (52.8%)		70 ± 0.0 (100%)		70 ± 0.0 (100%)		
12.5 μM			70 ± 0.0 (100%)			70 ± 0.0 (100%)			70 ± 0.0 (100%)		50 ± 0.0 (71.4%)	
6.25 μM			70 ± 0.0 (100%)			70 ± 0.0 (100%)			70 ± 0.0 (100%)			
**PBT5**												
200 μM	70 ± 0.0 (100%)			70 ± 0.0 (100%)			70 ± 0.0 (100%)			70 ± 0.0 (100%)		
100 μM	70 ± 0.0 (100%)			70 ± 0.0 (100%)			70 ± 0.0 (100%)			70 ± 0.0 (100%)		
50 μM		35 ± 0.0 (50%)	35 ± 0.0 (50%)		70 ± 0.0 (100%)		70 ± 0.0 (100%)			70 ± 0.0 (100%)		
25 μM			70 ± 0.0 (100%)			70 ± 0.0 (100%)			70 ± 0.0 (100%)		70 ± 0.0 (100%)	
12.5 μM			70 ± 0.0 (100%)			70 ± 0.0 (100%)			70 ± 0.0 (100%)		70 ± 0.0 (100%)	
6.25 μM			70 ± 0.0 (100%)			70 ± 0.0 (100%)			70 ± 0.0 (100%)			70 ± 0.0 (100%)
**PBT6**												
200 μM	70 ± 0.0 (100%)			70 ± 0.0 (100%)			70 ± 0.0 (100%)			70 ± 0.0 (100%)		
100 μM	70 ± 0.0 (100%)			70 ± 0.0 (100%)			70 ± 0.0 (100%)			70 ± 0.0 (100%)		
50 μM		48 ± 0.0 (58.6%)	22 ± 0.0 (31.4%)	70 ± 0.0 (100%)			70 ± 0.0 (100%)			70 ± 0.0 (100%)		
25 μM			70 ± 0.0 (100%)		18 ± 2.0 (25.7%)	52 ± 5.0 (74.3%)	70 ± 0.0 (100%)			70 ± 0.0 (100%)		
12.5 μM			70 ± 0.0 (100%)			70 ± 0.0 (100%)			70 ± 0.0 (100%)		55 ± 0.0 (78.6%)	15 ± 0.0 (21.4%)
6.25 μM			70 ± 0.0 (100%)			70 ± 0.0 (100%)			70 ± 0.0 (100%)			70 ± 0.0 (100%)

Control 1: Schistosomula incubated only in supplemented RPMI. Control 2: Schistosomula incubated in RPMI supplemented with 1.0% DMSO. Score 3: Typical movements along the body; peristalsis of internal organs and suckers adhered to the sides and bottom of the culture plate. Score 1.5: Movement only in the extremities (posterior and/or anterior). Score 0: Complete absence of movements and tegument with or without color change.

**Table 2:** Motility scores of young worms treated with praziquantel (PZQ) or thiazole compounds (**PBT2**, **PBT5**, or **PBT6**) after 3, 6, 12, and 24 h of incubation.

Experimental Groups	Mean ± Standard Deviation (SD) and Percentage (%) of Young Worms in Motility Scores After Incubation																
	3 h				6 h					12 h				24 h			
	0	1	2	3	0	1	2	3		0	1	2	3	0	1	2	3
Control 1				30 ± 0.0 (100%)				30 ± 0.0 (100%)					30 ± 0.0 (100%)				30 ± 0.0 (100%)
Control 2				30 ± 0.0 (100%)				30 ± 0.0 (100%)					30 ± 0.0 (100%)				30 ± 0.0 (100%)
PZQ																	
10 µM		30 ± 0.0 (100%)				30 ± 0.0 (100%)					30 ± 0.0 (100%)				30 ± 0.0 (100%)		
**PBT2**																	
200 µM	30 ± 0.0 (100%)				30 ± 0.0 (100%)					30 ± 0.0 (100%)				30 ± 0.0 (100%)			
100 µM	30 ± 0.0 (100%)				30 ± 0.0 (100%)					30 ± 0.0 (100%)				30 ± 0.0 (100%)			
50 µM		18 ± 4.0 (60%)	12 ± 3.0 (40%)			27 ± 2.5 (90%)	3 ± 0.0 (10%)				30 ± 0.0 (100%)			30 ± 0.0 (100%)			
25 µM				30 ± 0.0 (100%)			21 ± 2.0 (70%)	9 ± 2.0 (30%)				30 ± 0.0 (100%)				30 ± 0.0 (100%)	
12.5 µM				30 ± 0.0 (100%)				30 ± 0.0 (100%)					30 ± 0.0 (100%)				30 ± 0.0 (100%)
6.25 µM				30 ± 0.0 (100%)				30 ± 0.0 (100%)					30 ± 0.0 (100%)				30 ± 0.0 (100%)
**PBT5**																	
200 µM	30 ± 0.0 (100%)				30 ± 0.0 (100%)					30 ± 0.0 (100%)				30 ± 0.0 (100%)			
100 µM	30 ± 0.0 (100%)				30 ± 0.0 (100%)					30 ± 0.0 (100%)				30 ± 0.0 (100%)			
50 µM			24 ± 4.0 (80%)	6 ± 1.0 (20%)			30 ± 0.0 (100%)				19 ± 3.5 (66.33%)	11 ± 2.0 (36.66%)		30 ± 0.0 (100%)			
25 µM				30 ± 0.0 (100%)				30 ± 0.0 (100%)									30 ± 0.0 (100%)
12.5 µM				30 ± 0.0 (100%)				30 ± 0.0 (100%)									30 ± 0.0 (100%)
6.25 µM				30 ± 0.0 (100%)				30 ± 0.0 (100%)									30 ± 0.0 (100%)
**PBT6**																	
200 µM	30 ± 0.0 (100%)				30 ± 0.0 (100%)					30 ± 0.0 (100%)				30 ± 0.0 (100%)			
100 µM	30 ± 0.0 (100%)				30 ± 0.0 (100%)					30 ± 0.0 (100%)				30 ± 0.0 (100%)			
50 µM		30 ± 0.0 (100%)			30 ± 0.0 (100%)					30 ± 0.0 (100%)				30 ± 0.0 (100%)			
25 µM				30 ± 0.0 (100%)			30 ± 0.0 (100%)				15 ± 0.0 (50%)	15 ± 0.0 (50%)			30 ± 0.0 (100%)		
12.5 µM				30 ± 0.0 (100%)				30 ± 0.0 (100%)					30 ± 0.0 (100%)				30 ± 0.0 (100%)
6.25 µM				30 ± 0.0 (100%)				30 ± 0.0 (100%)					30 ± 0.0 (100%)				30 ± 0.0 (100%)

Control 1: Young worms incubated only in supplemented RPMI. Control 2: Young worms incubated in RPMI supplemented with 1.0% DMSO. Score 3: Typical movements along the body; peristalsis of internal organs and suckers adhered to the sides and bottom of the culture plate. Score 2: Reduced movements along the body and reduced peristalsis. Score 1: Movement only in the extremities (posterior and/or anterior). Score 0: Complete absence of movements and tegument with or without color change.

**Table 3:** Cytotoxicity (CC_50_: inhibits cell growth by 50%), lethal concentration antiparasitic (CL_50_: 50% lethality of worms) and selectivity index (SI) promoted by thiazole compounds (**PBT2**, **PBT5**, and **PBT6**).

Compounds	Splenocytes CC_50_ (μM)	Peritoneal Macrophages CC_50_ (μM)	*S. masoni* (CL_50_ μM)		SI Splenocytes		SI Peritoneal Macrophages	
			Schistosomes	Young	Schistosomes	Young	Schistosomes	Young
**PBT2**	193.9 ± 8.4	277.5 ± 11.6	27.84 ± 3.4	32.85 ± 3.8	6.96	5.9	9.96	8.44
**PBT5**	204.2 ± 12.6	281.9 ± 9.4	30.89 ± 2.8	34.92 ± 3.2	6.61	5.84	9.12	8.07
**PBT6**	297.3 ± 15.8	>400	15.33 ± 2.2	27.76 ± 2.5	19.39	10.7	>26	>14.4
PZQ	3.2 ± 0.8	>400	>10	>10	<0.32	<0.32	>40	>40

Note: Results expressed as mean ± standard deviation.

**Table 4:** Effects of thiazole compounds (**PBT2**, **PBT5**, or **PBT6**) and praziquantel (PZQ) on the parasite load of worms (total and females) and eggs in the liver and intestinal tissues of mice infected with *S. mansoni*, euthanized 55 days after infection.

Groups	Average Worm Burden				Number of Eggs/g Tissue × 10^3^			
	Total	Reduction (%)	Female	Reduction (%)	Hepatic	Reduction (%)	Intestinal	Reduction (%)
Control 1	59.89 ± 6.9	-	27.9 ± 3.9	-	7.12 ± 0.87	-	11.58 ± 0.66	-
Control 2	61.45 ± 6.17	-	28.3 ± 4.08	-	7.45 ± 1.22	-	12.16 ± 2.11	-
PZQ								
50 mg/kg	0.55 ± 0.73	99.08	0.44 ± 0.53	98.42	0.88 ± 0.85	87.64	2.11 ± 0.49	81.78
**PBT2**								
200 mg/kg	22.57 ± 6.92 ***	62.31	11.86 ± 2.41 ***	57.49	3.86 ± 0.73 ***	45.79	4.9 ± 0.14 ***^,e^	57.68
100 mg/kg	32.67 ± 11.81 ***	45.44	15.17 ± 6.05 ***	45.62	4.11 ± 0.81 ***	42.28	7.11 ± 0.52 ***^,a^	38.6
50 mg/kg	36.14 ± 5.52 ***^,a^	39.65	16.13 ± 2.75 ***	42.18	4.53 ± 0.74 ***	36.38	6.92 ± 0.45 ***^,a^	40.24
**PBT5**								
200 mg/kg	22.86 ± 4.59 ***	61.83	9.71 ± 2.21 ***	65.19	2.89 ± 0.47 ***	59.41	4.46 ± 0.39 ***	61.5
100 mg/kg	25.29 ± 6.18 ***	57.77	12.14 ± 2.61 ***	56.48	3.15 ± 0.38 ***^,b^	55.75	5.28 ± 0.7 ***^,b^	54.4
50 mg/kg	53.14 ± 8.95 ***^,b^	11.27	25.57 ± 5.06 ^b^	8.35	6.5 ± 0.54 ^b^	8.7	10.7 ± 0.1 ^b^	7.6
**PBT6**								
200 mg/kg	20.57 ± 6.82 ***	65.65	12.0 ± 3.1 ***^,^	56.98	3.89 ± 0.75 ***	45.4	5.1 ± 0.79 ***	55.96
100 mg/kg	33.14 ± 6.74 ***^,c^	44.66	19.50 ± 3.6 ***^,c^	30.11	4.67 ± 0.61 ***	34.41	8.15 ± 0.74 ***^,d^	29.62
50 mg/kg	37.83 ± 3.25 ***^,d^	36.83	18.29 ± 2.81 ***^,d^	34.44	4.41 ± 0.43 ***^,c^	38.06	8.2 ± 0.11 ***^,d^	29.2

Control 1 = infected animals that only sterile saline solution. Control 2 = infected animals that received 1% Cremophor^®^ in sterile saline solution. *** *p* < 0.001 significant difference compared to the control1 group. ^a^ *p* < 0.01 significant difference compared to **PBT2** at 200 mg/kg. ^b^ *p* < 0.001 significant difference compared to **PBT5** at 200 and 100 mg/kg. ^c^ *p* < 0.01, ^d^ *p* < 0.001 significant difference compared to **PBT6** at 200 mg/kg. ^e^ *p* < 0.001 significant difference compared to **PBT2** at 200 and 50 mg/kg.

**Table 5:** Effect of thiazole compounds (**PBT2**, **PBT5**, and **PBT6**) and praziquantel (PZQ) on the oogram of mice infected with *S. mansoni* when administered from the 45th to the 49th day post-infection and euthanized on the 55th day of infection.

Groups	% of Eggs per Developmental Stage		
	Immature	Mature	Dead
Control 1	57.2 ± 8.52	38.0 ± 7.03	4.8 ± 1.64
Control 2	55.4 ± 6.31	39.8 ± 4.86	4.8 ± 1.92
PZQ			
50 mg/kg	0.0 ± 0.0 ***	4.2 ± 0.83 ***	95.8 ± 0.83 ***
**PBT2**			
200 mg/kg	15.4 ± 6.38 ***	28.4 ± 3.21 ***	56.2 ± 7.08 ***
100 mg/kg	27.6 ± 3.36 ***^,a^	38.6 ± 4.15 ^b^	33.8 ± 5.02 ***^,a^
50 mg/kg	39.4 ± 5.17 ***^,b^	43.8 ± 5.21 ^a^	16.8 ± 3.63 ***^,a^
**PBT5**			
200 mg/kg	17.0 ± 4.0 ***	29.8 ± 4.47 ***	53.2 ± 2.16 ***
100 mg/kg	29.4 ± 3.78 ***^,b^	35.8 ± 5.16	34.8 ± 6.14 ***^,a^
50 mg/kg	55.4 ± 2.51 ^a^	39.0 ± 2.23 ^a^	5.6 ± 1.51 ^a^
**PBT6**			
200 mg/kg	18.0 ± 2.34 ***	22.6 ± 8.4 ***	59.4 ± 6.5 ***
100 mg/kg	26.4 ± 3.21 ***^,b^	36.0 ± 2.55 ^a^	37.6 ± 4.15 ***^,a^
50 mg/kg	39.6 ± 4.72 ***^,a^	37.6 ± 4.33 ^a^	22.8 ± 4.65 ***^,a^

Control 1 = infected animals that received only sterile saline solution. Control 2 = infected animals that received 1% Cremophor^®^ in sterile saline solution. *** *p* < 0.001 significant difference compared to the control 1 and 2 groups. ^a^ *p* < 0.001, ^b^ *p* < 0.01 significant intragroup difference with 200 mg/kg at the same developmental stage.

## Data Availability

The original contributions presented in this study are included in the article. Further inquiries can be directed to the corresponding author.
